# Supplementary material for: Development of a Broad-Spectrum Antiserum against Cobra Venoms Using Recombinant Three-Finger Toxins
Source: Toxins (Basel). 2021 Aug 10;13(8):556. doi: 10.3390/toxins13080556 (PMC8402450; doi:10.3390/toxins13080556)
Supplement: Supplementary file 1 [file toxins-13-00556-s001.zip › toxins-1315811-supplementary.pdf]

toxins

Supplementary Materials: Development of a Broad-Spectrum Antiserum against Cobra Venoms Using Recombinant Three-Finger Toxins

Bing-Sin Liu, Bo-Rong Jiang, Kai-Chieh Hu, Chien-Hsin Liu, Wen-Chin Hsieh, Min-Han Lin and Wang-Chou Sung

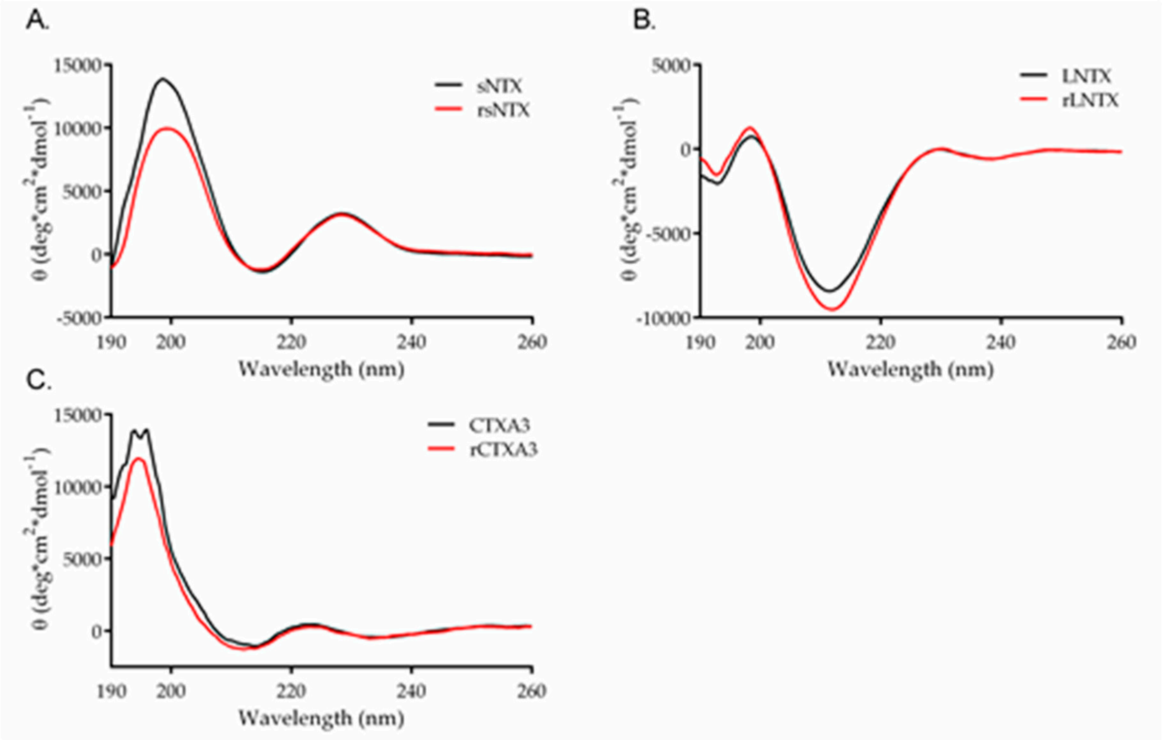

Figure S1. The CD spectra of native and recombinant (**A**) sNTX, (**B**) LNTX, and (**C**) CTXA3. The spectra of native toxins purified from venoms are shown in black lines; the spectra of recombinant toxins are shown in red lines.

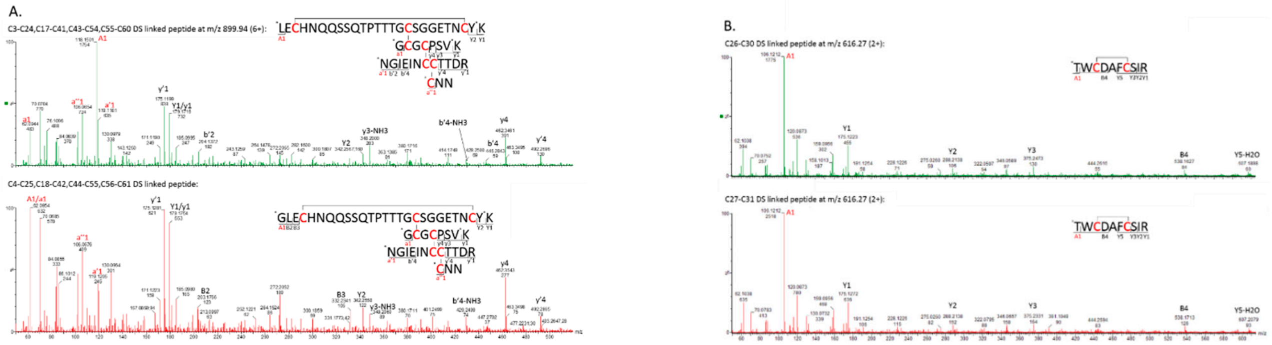

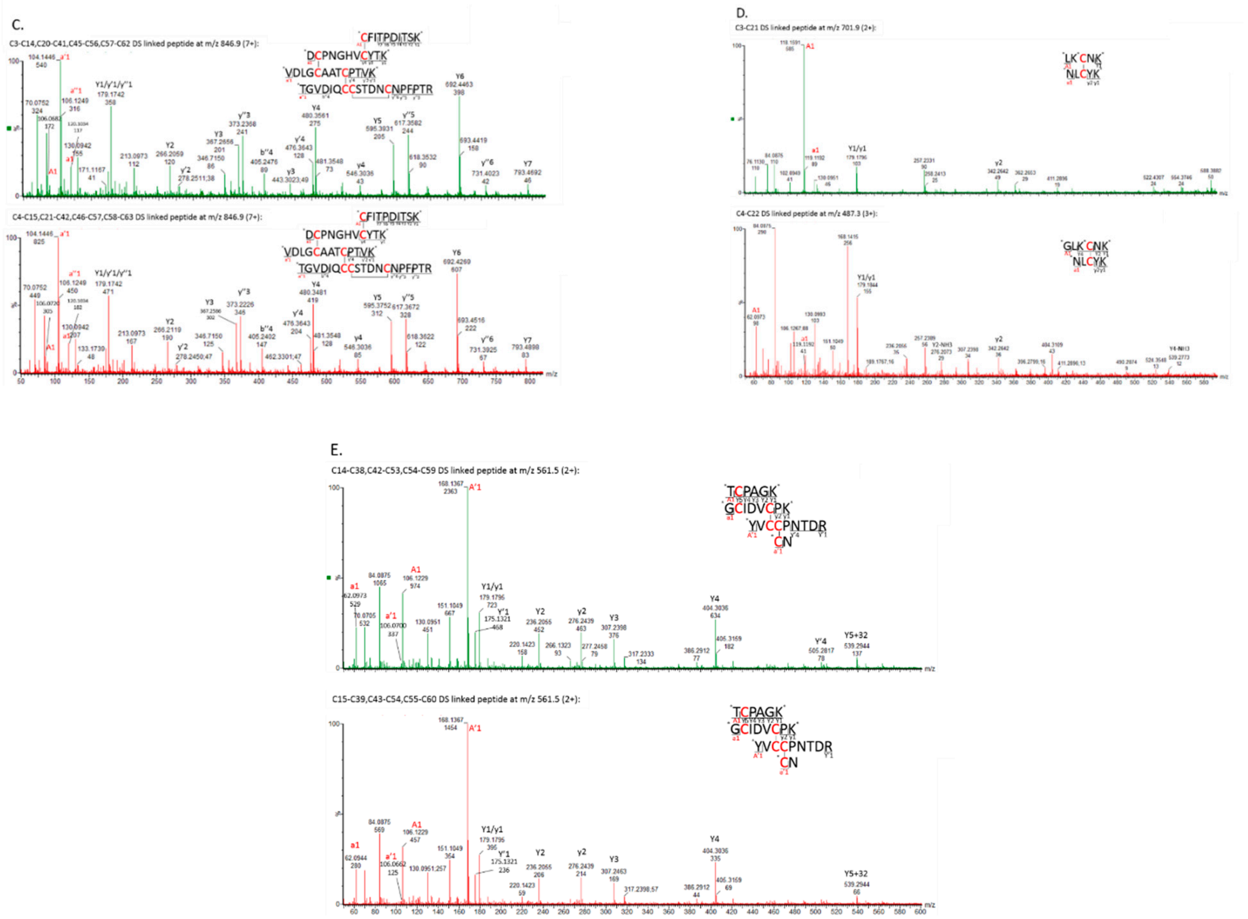

Figure S2. Identification of linkage sites of disulfide bonds by MS analysis. Comparative MS/MS spectra of the disulfide bond-containing peptides derived from the tryptic digests of native (green) or recombinant (red) (**A**) sNTX, (**B**,**C**) LNTX, and (**D**,**E**) CTXA3. CID fragment ions (a1/a′1/A1/A′1 and y/y1/Y/Y′ ions) are annotated. Asterisks (*) indicate the sites of dimethyl (d2) labeling in the peptides.

A.

Species	Ac No [a]	Ratio [b]	Sequence (1–71)	Identity/Similarity [c] (%)	Ref.
N. kaouthia<br>*N. siamemsis*	P01391	33.3<br>22.4	IRCFITPDITSKDCPNGHVCYTKTWCDAFCSIRGKRVDLGCAATCPTVKTGVDIQCCSTDNCNPFPTRKRP	100/100	[32]<br>[8]
N. sumatrana	O42257	8.7	IRCFITPDVTSTDCPNGHVCYTKTWCDGFCSSRGRRVELGCAATCPTVKPGVDIQCCSTDNCNPFPTRP--	90/94	[33]
N. naja	P25668	15.7	IRCFITPDITSKDCPNGHVCYTKTWCDGFCSIRGKRVDLGCAATCPTVRTGVDIQCCSTDDCDPFPTRKRP	94/98	[34]
N. melanoleuca	P01388	14.6	IRCFITPDVTSQICADGHVCYTKTWCDNFCASRGKRVDLGCAATCPTVKPGVNIKCCSTDNCNPFPTRNRP	83/91	[35]

B.

Species	Ac No [a]	Ratio [b]	Sequence (1–62)	Identity/Similarity [c] (%)	Ref.
N. atra	P60770	12.1	LECHNQQSSQTPTTTGCSGGETNCYKKRWRDHRGYRTERGCGCPSVKNGIEINCCTTDRCNN	100/100	[13]
N. kaouthia	P60771	2.3-6.6	LECHNQQSSQTPTTTGCSGGETNCYKKRWRDHRGYRTERGCGCPSVKNGIEINCCTTDRCNN	100/100	[32]
N. kaouthia<br>*N. naja*<br>*N. siamensis*	P59276	0.3-4.1<br>3.6<br>1.7	LECHNQQSSQAPTTKTCSG-ETNCYKKWWSDHRGTIIERGCGCPKVKPGVNLNCCRTDRCNN	76/79	[32]<br>[34]<br>[8]
N. naja	P01427	0.4	LECHNQQSSQPPTTKTCSG-ETNCYKKWWSDHRGTIIERGCGCPKVKPGVNLNCCRTDRCNN	76/79	[34]
N. siamensis	P59275	2.8	LECHNQQSSQTPTTKTCSG-ETNCYKKWWSDHRGTIIERGCGCPKVKPGVNLNCCRRDRCNN	76/79	[8]
	P60773	-	LECHNQQSSQAPTTKTCSG-ETNCYKKWWSDHRGTIIERGCGCPKVKPGVKLNCCRTDRCNN	76/80	
N. sputatrix	Q9PSN6	2.3	LECHDQQSSQTPTTTGCSGGETNCYKKRWRDHRGYRTERGCGCPSVKNGIEINCCTTDRCNN	98/100	[36]
	AAA66025	1.5	LECHDQQSSETPTTTGCSGGETNCYKKSWRDHRGYRIERGCGCPSVKKGIEINCCTTDRCNN	92/95	[36]
N. mossambica	P01420	< 5	MICYKQQSLQFPITTVCPG-EKNCYKKQWSGHRGTIIERGCGCPSVKKGIEINCCTTDKCNR	69/75	[37]
N. melanoleuca	P01424	9.4	MECHNQQSSQPPTTKTCPG-ETNCYKKQWSDHRGTIIERGCGCPSVKKGVKINCCTTDRCNN	77/83	[35]

C.

Species	Ac No [a]	Ratio [b]	Sequence (1–60)	Identity/Similarity [c] (%)	Ref.
N. atra	P60301	18.8	LKCNKLVPLFYKTCPAGKNLCYKMFMVATPKVPVKRGCIDVCPKSSLLVKYVCCNTDRCN	100/100	[13]
	P60304	18.2	LKCNKLIPIASKTCPAGKNLCYKMFMMSDLTIPVKRGCIDVCPKNSLLVKYVCCNTDRCN	82/92	[13]
	P01442	3.0	LKCNKLVPLFYKTCPAGKNLCYKMFMVSNLTVPVKRGCIDVCPKNSALVKYVCCNTDRCN	91/93	[13]
	P01443	6.6	RKCNKLVPLFYKTCPAGKNLCYKMFMVSNLTVPVKRGCIDVCPKNSALVKYVCCNTDRCN	88/92	[26]
N. kaouthia	P01445	20.1	LKCNKLIPLAYKTCPAGKNLCYKMFMVSNKTVPVKRGCIDVCPKNSLLVKYVCCNTDRCN	88/93	[32]
	P01446	17.1	LKCNKLIPLAYKTCPAGKNLCYKMFMVSNKTVPVKRGCIDACPKNSLLVKYVCCNTDRCN	87/91	[32]
	P60305	10.6	LKCNKLIPIASKTCPAGKNLCYKMFMMSDLTIPVKRGCIDVCPKNSLLVKYVCCNTDRCN	82/91	[32]
	Q9W6W9	2.0	RKCNKLVPLFYKTCPAGKNLCYKMFMVSNLTVPVKRGCIDVCPKSSLLVKYVCCNTDRCN	92/93	[32]
	O73857	8.6	LKCNKLVPLFYKTCPAGKNLCYKMFMVSNLTVPVKRGCIDVCPKNSALVKYVCCNTDRCN	90/93	[32]
N. sputatrix	P07525	12.8	LKCNKLVPLFYKTCPAGKNLCYKMFMVSNKMVPVKRGCIDVCPKSSLLVKYVCCNTDRCN	93/95	[36]
	P60311	22.7	DKCNKLVPLFYKTCPAGKNLCYKMFMVSDLTVPVKRGCIDVCPKNSALVKYVCCNTDRCN	90/93	[36]

Figure S3. Sequence alignment of the major (**A**) LNTXs, (**B**) sNTXs, and (**C**) CTXs identified in cobra snake venoms. The percentage of amino acids that are identical or similar at the same position compared to the sequence of P01391 (for LNTXs), P60770 (for sNTXs), or P60301 (for CTXs). Conserved amino acids identical in all sequences of the same subfamily are shown in red with bright yellow background; similar amino acids are shown in black with blue or green background. The accession numbers of proteins were from the UniProt database. The percentage (%) of relative abundance in the corresponding venoms was reported in the literatures.

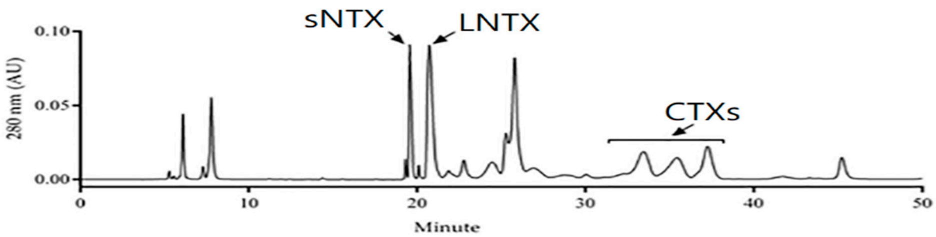

Figure S4. RP-HPLC elution chromatogram of the cobra venom mixture for rabbit immunization experiment. The ratio of sNTX:LNTX:CTXs in the venom mixture was estimated to be 1:1:3 based on the peak areas.

Table S1. Identification of *N. atra and N. kaouthia* venom components in the HPLC fractions using MS.

Peak [1]	Protein Identity	AC no. [2]	Significant Peptide	Observed Ion (m/z)	MS Score	Toxin Family
**a**	Cobrotoxin	P60770	R.GCGCPSVK.N<br>K.NGIEINCCTTDR.C	432.6748 (2+)<br>726.7258 (2+)	69	3FTX
**b**	Acidic phospholipase A2	A4FS04	-.NLYQFK.N<br>K.GGSGTPVDDLDR.C	406.7119 (2+)<br>594.7706 (2+)	89	PLA2
**c**	Cytotoxin 1	P60304	K.MFMMSDLTIPVK.R	706.8398 (2+)	375	3FTX
	Cytotoxin 3	P60301	K.MFMVATPK.V<br>K.LVPLFYK.T	462.7221 (2+)<br>440.2524 (2+)	101	
	Cytotoxin 5	P07525	K.MVPVKR.G	373.2054 (2+)	46	
**d**	Cobrotoxin-b	P59275	K.TCSGETNCYK.K<br>K.WWSDHR.G<br>K.VKPGVNLNCCR.R<br>-.LECHNQQSSQTPTTK.T<br>-.LECHNQQSSQTPTT-KTCSGETNCYK.K	610.2822 (2+)<br>443.7183 (2+)<br>439.5856 (3+)<br>879.9451 (2+)<br>987.1167 (3+)	197	3FTX
	Cobrotoxin-c	P59276	-.LECHNQQSSQAPTTK.T	576.9522 (2+)	149	3FTX
	Alpha-elapitoxin-Nk2a	P01391	R.CFITPDITSK.D<br>K.TWCDAFCSIR.G<br>R.VDLGCAATCPTVK.T	591.3514(2+)<br>658.3432 (2+)<br>696.3925 (2+)	98	3FTX
	Probable weak neuro-toxin NNAM1	Q9YGI2	R.GCADTCPVR.K<br>R.EIVECCSTDK.C	518.2423 (2+)<br>620.8072 (2+)	70	3FTX
	Weak neurotoxin 6	P29180	-.LTCLICPEK.Y	567.3386 (2+)	68	3FTX
**e**	Alpha-elapitoxin-Nk2a	P01391	R.CFITPDITSK.D<br>K.TWCDAFCSIR.G<br>R.VDLGCAATCPTVK.T	591.3469 (2+)<br>658.3644 (2+)<br>696.3752 (2+)	124	3FTX
**f**	Acidic phospholipase A2 2	P15445	K.ISGCWPYFK.T<br>R.GGSGTPVDDLDR.C<br>R.SWWDFADYGCYCGR.G<br>K.NMIKCTVPSR.S [3]<br>R.LAAICFAGAPYNDNNYNIDLK.A	579.3079 (2+)<br>594.8204 (2+)<br>921.9120 (2+)<br>611.3373 (2+)<br>786.4366 (3+)	309	PLA_2
	Acidic phospholipase A2 D	Q9I900	K.NMIQCTVPNR.S<br>R.GGSGTPVDDLDR.C<br>R.CWPYFK.T	624.8400 (2+)<br>594.8148 (2+)<br>450.7263 (2+)	295	PLA_2
	Acidic phospholipase A2 2	P00597	R.LAAICFAGAPYNNNNYNIDLK.A<br>K.GGSGTPVDDLDR.C	1178.6711 (2+)<br>594.8255 (2+)	268	PLA_2
	Acidic phospholipase A2 DE-II	P00600	R.GGSGTPVDDLDR.C<br>K.NMIQCTVPNR.S	594.8200 (2+)<br>624.8298 (2+)	200	PLA_2
	Venom nerve growth factor 2	Q5YF89	K.TTATDIK.G<br>K.QYFFETK.C<br>K.ALTMEGNQASWR.F<br>K.GNTVTVMENVNLDNK.V	375.2329 (2+)<br>481.7730 (2+)<br>682.3720 (2+)<br>824.4734 (2+)	131	VGF
**g**	Thaicobrin	P82885	R.FDGSPCVLGSPGFR.S<br>K.TVENVGVSQVAPDNPER.F<br>K.ADVTFDSNTAFESLVVSPDK.K	748.4003 (2+)<br>906.0082 (2+)<br>1071.5872 (2+)	282	Vespryn
	Cytotoxin 1	P60305	K.LIPIASK.T<br>R.GCIDVCPK.N<br>K.YVCCNTDR.C<br>K.MFMMSDLTIPVK.R	371.2701 (2+)<br>474.7489 (2+)<br>544.2528 (2+)<br>722.8949 (2+)	236	3FTX
	Cytotoxin 5	P24779	K.LIPLAYK.T<br>K.MFMVAAPK.V<br>R.GCIDACPK.N	409.2871 (2+)<br>455.7598 (2+)<br>460.7300 (2+)	93	3FTX

	Cytotoxin 4	P60303	K.MFMVATPK.V	478.7623 (2+)	45	3FTX
	Cytotoxin 2	Q9DGH9	K.IFMVATPK.V	461.7617 (2+)	57	3FTX
	Cytotoxin 5	P07525	K.MFMVSNKMVPVK.R	713.8943 (2+)	57	3FTX
**h**	Cysteine-rich venom protein natrin-1	Q7T1K6	K.LTNCDSLLK.Q<br>R.VSPTASNMLK.M<br>K.EIVDLHNSLR.R<br>R.RVSPTASNMLK.M<br>K.QSSCQDDWIK.S<br>K.QKEIVDLHNSLR.R<br>K.MEWYPEAASNAER.W<br>R.TWTEIIHLWHDEYK.N<br>R.VLEGIQCGESIYMSSNAR.T	532.2916 (2+)<br>532.3129 (2+)<br>399.2381 (3+)<br>610.3337 (2+)<br>633.8123 (2+)<br>484.6273 (3+)<br>785.2921 (2+)<br>624.3440 (3+)<br>1015.5442 (2+)	674	CRISP
	Cysteine-rich venom protein annuliferin-a (Fragment)	P0DL14	-.NVDFNSESTR.R	584.8115 (2+)	152	CRISP
	Cysteine-rich venom protein ophanin	Q7ZT98	K.QSSCQDEWIK.S	640.8143 (2+)	119	CRISP
	Cobra venom factor	Q91132	R.IDVPLQIEK.A	527.8409 (2+)	117	CVF

[1] The peak refers to the labeling in Figure 3. [2] AC is the accession number in Uniprot database. [3] The underline of methionine (M) means it was oxidized. Abbreviations: SVMP indicates snake venom metalloproteinase. CRISP is cysteine-rich secretory protein. 3FTX means three finger toxins.
